# Promoting chlamydia screening with posters and leaflets in general practice - a qualitative study

**DOI:** 10.1186/1471-2458-9-383

**Published:** 2009-10-12

**Authors:** Elaine Freeman, Rebecca Howell-Jones, Isabel Oliver, Sarah Randall, William Ford-Young, Philippa Beckwith, Cliodna McNulty

**Affiliations:** 1 Gloucestershire Research & Development Support Unit, Gloucestershire Hospitals NHS Foundation Trust, Great Western Road, Gloucester, UK; 2 Health Protection Agency, Centre for Infections, HIV and STI Department Colindale, London, UK; 3 Health Protection Agency South West, The Wheelhouse, Bond's Mill, Stonehouse, UK; 4 Ella Gordon Unit, St Mary's Hospital, Portsmouth, UK; 5 Broken Cross Surgery, Waters Green Medical Centre, Macclesfield, UK; 6 Cardiff University, Cardiff, UK; 7 Health Protection Agency Primary Care Unit, Microbiology Department, Gloucestershire Royal Hospital, Great Western Road, Gloucester GL1 3NN, UK

## Abstract

**Background:**

General practice staff are reluctant to discuss sexual health opportunistically in all consultations. Health promotion materials may help alleviate this barrier. Chlamydia screening promotion posters and leaflets, produced by the English National Chlamydia Screening Programme (NCSP), have been available to general practices, through local chlamydia screening offices, since its launch. In this study we explored the attitudes of general practice staff to these screening promotional materials, how they used them, and explored other promotional strategies to encourage chlamydia screening.

**Methods:**

Twenty-five general practices with a range of screening rates, were purposively selected from six NCSP areas in England. In focus groups doctors, nurses, administrative staff and receptionists were encouraged to discuss candidly their experiences about their use and opinions of posters, leaflets and advertising to promote chlamydia screening. Researchers observed whether posters and leaflets were on display in reception and/or waiting areas. Data were collected and analysed concurrently using a stepwise framework analytical approach.

**Results:**

Although two-thirds of screening practices reported that they displayed posters and leaflets, they were not prominently displayed in most practices. Only a minority of practices reported actively using screening promotional materials on an ongoing basis. Most staff in all practices were not following up the advertising in posters and leaflets by routinely offering opportunistic screening to their target population. Some staff in many practices thought posters and leaflets would cause offence or embarrassment to their patients. Distribution of chlamydia leaflets by receptionists was thought to be inappropriate by some practices, as they thought patients would be offended when being offered a leaflet in a public area. Practice staff suggested the development of pocket-sized leaflets.

**Conclusion:**

The NCSP should consider developing a range of more discrete but eye catching posters and small leaflets specifically to promote chlamydia screening in different scenarios within general practice; coordinators should audit their use. Practice staff need to discuss, with their screening co-ordinator, how different practice staff can promote chlamydia screening most effectively using the NCSP promotional materials, and change them regularly so that they do not loose their impact. Education to change all practice staff's attitudes towards sexual health is needed to reduce their worries about displaying the chlamydia materials, and how they may follow up the advertising up with a verbal offer of screening opportunistically to 15-24 year olds whenever they visit the practice.

## Background

Genital chlamydia is the most common sexually transmitted disease in Europe [1,2]. The English National Chlamydia Screening Programme (NCSP), first introduced in 2003, offers opportunistic screening to sexually active young people aged 15-24 to reduce prevalence of chlamydia to prevent ectopic pregnancy, pelvic inflammatory disease and infertility in men and women [3]. General practice is widely used by young people [4] and, therefore, provides an opportunity to raise awareness of, and provide, chlamydia screening.

Health promotion posters and leaflets produced by the Department of Health and NCSP have been available to general practice through local chlamydia screening offices since the launch of the NCSP. The NCSP leaflet was first produced in April 2003 and was based on the leaflet originally used in the chlamydia screening pilot based in several health care settings including general practice [5]. It was produced by the National Chlamydia Screening Programme Steering Group, which had GP representation, and was reviewed by other stakeholders. The leaflet was designed using a Department of Health (DH) format and to fit in with the ongoing DH Sexual Health Awareness campaign. It was designed to be used by all clinicians as part of the screening consent procedure. The posters were produced by the DH as part of a Sex Lottery campaign to be used in a range of Health Care settings. Additionally, many local areas produce their own promotional information materials. This reflects the devolved nature of the NSCP with much of the funding and responsibility for publicity and promotion at local level [3].

We and others have found that many general practice staff admitted that they are reluctant to discuss sexual health opportunistically in all consultations [6-10]. Health promotion materials may help alleviate this barrier but there are no published studies of how the NCSP promotional materials are being used in England. The objective of this study was to explore the attitudes of general practice staff to health promotional materials aimed at increasing uptake of chlamydia screening, how staff used them, and explore other promotional strategies to encourage chlamydia screening. This was part of a larger qualitative study exploring general practice staffs' knowledge of the chlamydia screening programme and strategies they have used or suggest to encourage increased chlamydia screening within the general practice setting.

## Methods

Disaggregate data from the Health Protection Agency (HPA) Centre for Infections [11] were used to identify and rank general practices by their chlamydia screening rates of their 16-24 year old target population. So that we could obtain the opinions of a wide range of general practices and staff, 25 high and low screening general practices were selected, using criterion based (purposive) sampling [12], from six NCSP areas in England who were encouraging screening within primary care. A member of the research team approached the practices by telephone and letter. We conducted eight focus groups with high screening practices (defined as those screening greater than 10% of their 16-24 year old target population), ten medium screening practices (between 3-10%) and 15 low screening practices (less than 3%).

Two high and six low screening practices declined to participate due to time pressures or staff shortages. Participating practices were visited between November 2005 and April 2007 and included those in both urban and rural locations, with a mix of social class and ethnic populations. Although researchers did not know the practice screening rates before each visit, it was difficult to blind them to whether the practice was a high or low screener, as this usually became quite apparent during the discussions. Doctors, nurses, administrative staff and receptionists were invited to participate in the focus group. The focus group schedule used open questions which encouraged respondents to discuss candidly their experiences of the chlamydia screening programme. As part of the focus group, we sought information about their use and opinions of posters, leaflets and advertising to promote chlamydia screening. Researchers observed whether posters and leaflets were on display in reception and/or waiting areas when they visited the practice and recorded field notes following each focus group. Data were collected and analysed using a stepwise framework analytical approach [13]. Focus groups were audio-taped then transcribed and checked for accuracy against the tapes. EF and CMcN used QSR NVivo software (QSR International PTY Ltd. Melbourne ) to identify codes, categories and themes from the data, using an inductive approach. This approach was used as we wanted to be open to using the depth and breadth of data collected to show the opinions and behaviour of the whole general practice team. Themes were then discussed at a project meeting and agreed by all the authors. If there were any disagreements, the text was re-examined and a consensus reached. Data collection and analysis occurred concurrently and we continued, through purposive sampling, to enrol and visit practices to enrich the data [12]. The transcripts were then re-analysed by EF, using word-search, to ensure that any themes within the framework were not missed. The relationship between the practice screening rates (high, medium and low) and the data was examined. Quotations chosen demonstrate the different categories of data. These quotes were chosen as they highlighted the diversity of opinion in both high and low screening practices and differences between them.

Ethical approval was obtained from the Multi-Research Ethics Committee for Scotland (No. 4/MRE10/41). Local research governance approval was obtained from the relevant Trusts. Information sheets were sent to the practices at least two weeks before the focus group and all staff gave informed written consent and were assured anonymity.

## Results

156 health care staff from 25 practices (participants per focus group 2-20; median 6) from urban and rural areas across England participated in the focus groups. These comprised 72 GPs, 46 nurses; eight practice managers; 23 receptionists/administrators and seven others.

### Use of chlamydia screening posters in general practices

#### Major themes

##### Use of posters

Two thirds (16) of all screening practices said they had posters advertising chlamydia in their practices, which were either on the doors of their consulting rooms, in their waiting rooms or in patients' lavatories.

*We also had *[chlamydia] *posters up around and also posters on our notice board outside and ... it's on our LED *[electronic sign in reception] *(Nurse FG14 medium screening practice)*

*There's *[general] *posters throughout the surgery isn't there and in the passageways especially down by the nurses end. I don't think the age group is on them. There was a *[chlamydia] *poster up, but it has been taken down. (Receptionist FG8 low screening practice)*

However, researchers observed that most posters displayed in general practices were aimed at elderly people or promoted immunisation; very few had chlamydia posters in communal areas.

*The difficulty with it, we tend to use the posters in the short term campaigns. We could do a campaign for an evening a month but then because of the wall space and everything we have to do. I mean we used to have lots of all sorts of *[different] *posters, it was too messy and too much information to read. (GP and nurse FG18 low screening practice)*

Many staff thought posters caused offence. Several practices had to deal with complaints from older people about posters advertising chlamydia screening in the waiting room or lavatories and patients had either removed the poster themselves or had asked for it to be taken down.

*We put up a poster on how to do it *[take a specimen] *in the toilets that got taken down*.

*We didn't have very much advertising *[about chlamydia] *because that actually upset quite a few patients. Especially the elderly, they were quite upset with our advertising. So we had to take it down and they said they didn't really want it in their face when they were sitting in the waiting room. We had quite a few people complain. We also had a piece on safe sex as well. And I think two people found that quite offensive, so we had to redo the advertising side*. (GP *FG2 medium screening practice respondent 1 & 3)*

One low screening practice said there had been posters in nurses' rooms but these posters had been taken down and not replaced when the practice was refurbished. Many practices were concerned that posters may lose their impact if left on display.

*My only problem is leaflets and posters go up and they become part of the scenery nobody takes any *[notice] *not a lot of impact. I think what we have to do would be *[for] *two weeks in a year, to have an impact on *[chlamydia], *have loads of posters up just for that *[time]. *(Practice nurse FG4 low screening practice)*

A few low screening practices reported other priorities for wall space and did not wish to prioritise one disease over another.

*We've got to be a bit sensitive. We've got the whole practice populations' needs *[to think about] *and I think we're not just here to deal with chlamydia we're here to deal with everything and things should be targeted equally. We've got a notice in each of our rooms on chlamydia so we have given it more space than some other diseases such as diabetes or asthma. We do try to give reasonably equal space don't we? (Practice nurse FG17 low screening practice)*

A few professionals in low screening practices admitted they had not seen NCSP posters and were unaware of where they could access chlamydia screening health promotional materials.

*I mean is it accessible *[to us], *or do we have to get our own literature? (Practice nurse FG11 high screening practice)*

### Use of chlamydia leaflets in general practices

#### Major themes

Most of the low screening practices had no chlamydia leaflets evidently on display when the researchers visited. Nineteen screening practices said they either had leaflets available in reception, waiting or consulting rooms, or included in self-sampling packs.

We leave leaflets in reception, and the primary care team produce a leaflet with all the different services that are available. Cards and a leaflet. We use it opportunistically for all our 16 year olds; that's part of the normal consultation. (Nurse FG3 high screening practice)

Displaying the leaflets in the waiting area for patients to help themselves was practices' main strategy for use.

*They *[the leaflets] *are quite good. There's leaflets that we keep in the waiting room and by the reception. (Practice nurse FG21, medium screening practice)*

[We have] *leaflets in waiting rooms (GP)*

...and we do have leaflets in our rooms, sometimes we have the little stand with 'what is chlamydia' and what you can do. (Practice nurse FG26 low screening practice)

#### Minor theme

*No recognition of leaflets*: A few health professionals admitted they couldn't remember seeing the NCSP leaflets.

*The big screen one *[see additional file 1] *I don't know what it is. Is that the coloured one?*

Could be?

....pass. (Nurse and two GPs FG 23 medium screening practice)

Facilitator: So what do you think about the chlamydia leaflets?

*I can't even remember what they look like*.

*Sorry I don't know what they look like*.

they're quite a colourful thing ..... to be honest to you no recollection that's it

(GP, nurse and receptionist FG24 low screening practice)

### Staff opinions about chlamydia screening leaflets

#### Major themes

##### Practices were enthusiastic about the leaflets

Most practices were generally enthusiastic about the NCSP leaflets. Fourteen practices thought that the NCSP leaflet was user friendly, featured different ethnic groups, was easy to read and very informative.

The leaflets for the patient are absolutely superb, very self-explanatory; they are quite small and very necessary because they're all waiting to go in to see the doctor. Its nice print, it's nicely put and nice to read it's so easy to explain. (Nurse FG11 high screening practice)

*In this area it is important to have more than white faces on a leaflet, that's gone down well*.

(Nurse FG15 low screening practice)

Several practices reported that they used the leaflets as part of their consent procedure for screening.

*I think it's excellent... it's very informative, but it's easy to read as well, which is quite important. We're supposed to *[give it to patients] *because its part of their consent procedure; consent is based on *[the] *leaflet. (GP FG3 high screening practice)*

##### Leaflets may cause offence

However, staff in several low and a few medium screening practices thought that giving young patients a leaflet would cause patients embarrassment, resentment or offence.

A patient might think are they picking on me? Why would they think I might have chlamydia? So I don't think it would go down so well here at the moment....sometimes they may be resentful as well. (Nurse FG15 low screening practice)

Of these a few low screening practices thought that the leaflets' style was condescending to young people and provided too much general information and omitted to advise on how often they should be screened.

They're fine, I think the actual main leaflet is fine, but I think the one with boys and girls on ...I think it's a bit condescending really, I think they're actually quite simplistic, so its easy to follow, but boys and girls, we're talking about people who are sexually active! I think it's a bit insulting putting boys on one and girls on the other personally. (Nurse FG17 low screening practice)

Other professionals in a few low screening practices thought that a chlamydia leaflet (especially the brightly coloured one) was a label for young people who may not wish to be seen with it or who would be offended by the suggestion that they may have chlamydia.

*I think one of the reasons why I don't always hand out leaflets, is that very often they *[the target group] *come in without a bag or anything and if they are walking around with this *[chlamydia leaflet] *its just the fact that it says 'chlamydia' everyone look at what I've *[got], *we're trying to normalise it but they often come in with just a jacket or a tiny purse*.

*That's a good point, quite a lot of them *[leaflets] *end up left on the desk and then we find them when they've gone; I wouldn't walk out of the GP surgery with that in my hand either... What is it saying and yet you've only come in to have your travel vacs? (GP and nurse FG01 high screening practice)*

Several thought leaflets should be concealed in envelopes. And a few other medium and low screening practices, following complaints from older people about chlamydia leaflets being highly visible in reception areas, had already put them in brown envelopes.

If the leaflet was in a brown envelope and you target an age group you could write along it hope you don't mind, but would you like to read this? I think they are a bit intrusive, because it would seem to some people that you were targeting them because you thought they might have a problem and I don't think that's right. (Receptionist FG8 low screening practice)

I think that's why we put them in brown envelopes because we wanted to reduce the older patients' concerns about it so we had to think about the other patients as well. Several, elderly ladies said it's too much in my face when I walk in, and I don't want to see this sort of thing. (FG2 medium screening practice)

##### Leaflets were too bulky

A few of all the practices thought chlamydia leaflets were bulky and did not fit easily into a jeans pocket, so leaflets were often left on the reception desk. These professionals suggested that the information could be given in a more concise form (bullet points) or be credit card sized and that the cover could be more discreet.

*I mean this is the only one I've got now... *[showing small card], *because it's not too bulky *[to go] *in a pocket. I agree totally absolutely fantastic, especially for younger people, because it doesn't contain *[too much], *you know *[you've] *got to sit there and read it. It's bullet points straight to the point facts given, no hassle with that. (Nurse FG9 high screening practice)*.

#### Minor themes

##### Leaflets should be translated

Some high and medium screening practices with high ethnic populations thought that the leaflet should be translated into different languages as patients may not understand written English. However some concern was expressed about the cost implications for the PCT, particularly in some locations where there were many different ethnic populations. One medium screening practice with a high ethnic population commented that pictorial leaflets should reflect the multi-cultural society that could be affected by chlamydia.

*Because people come with different languages, if you say some *[thing] *even if it's in simple English they would not understand the meaning of it. Preferably I would like to have them in as many languages as possible, but it's not really viable from the PCT point *[of view]. *They cannot have an enormous amount of languages just for chlamydia screening, there's other things as well. (GP FG25 medium screening practice)*

*It's all written in English. I've obviously got doubts about the value of translations with all different languages but I think they are required for our population*.

*...very expensive I think the cost to do it *[the leaflet translation]. *(2 GPs FG5 high screening practice)*

#### Other strategies for the use of chlamydia leaflets by general practices

One high screening practice reported that clinicians had given patients a chlamydia leaflet whenever they attended for any consultation and this had resulted in a good screening.

Making sure the leaflets are available and being a bit more pro-active about things......because we found that really worked before, we had a good uptake. (Nurse and GP FG11 high screening practice)

Furthermore, receptionists in a few high screening practices proactively gave patients in the at-risk age group a chlamydia leaflet to read when they booked in to see a doctor or nurse and encouraged patients to ask for a chlamydia screen.

The leaflets, we hand it over and say would you like to read this while you're sitting waiting to see your doctor, if you would like to take part, speak to the doctor when you go in. (Receptionist FG5 high screening practice)

##### Sending leaflets by post

A few practices thought that patients could be sent a leaflet, with an invitation to attend for chlamydia screening, by post and this would prompt patients to consult the practice for a screening test. These professionals thought that if patients were aware that they were all being targeted in this way it would encourage more young people to attend.

*Well we send out a letter to all the teenagers inviting them to the clinic and telling them what time, and it does say that we do tests for STIs. We're not specifically sending them information about chlamydia actually*.

*Maybe we should be enclosing a *[chlamydia] *information leaflet.... most people coming in we are mentioning it anyway. (2 GPs FG6 medium screening practice)*

*I think you've got to do it by post. You send a leaflet through the post saying that there is a *[chlamydia screening] *programme available; these are the people who are at risk; you may wish to consult; these are the complications if it's not treated; you may wish to consider it. (GP, FG26 low screening practice)*

A few medium screening practices thought that mobile phone texts may be a better way of encouraging young people to come forward for screening rather than sending letters to patients' homes.

*I don't know if this national advertising works*, [use] *the text via mobile, that would be a good point, they've all got mobile phones. (GP FG13 medium screening practice)*

### Other strategies for increasing awareness of chlamydia screening in general practice

#### Major themes

*National television advertising*: Many professionals in most of all the practices thought that NCSP needed a national advertising campaign either on prime time television or radio or included as an item in a leading soap opera. A few high and medium screening practices thought this high profile approach might be a better way to raise awareness of chlamydia screening with a multi-ethnic population.

*I mean what about a national campaign on prime time television, what I'm saying *[is a] *campaign on television that you can go to your GPs and be tested or *[get a] *self-test kit. Definitely I think better advertising would be a better use of time and energy really. (Nurse FG13 medium screening practice)*

*What about a national campaign *[on] *prime time television. (Nurse FG4 low screening)*

I think rather than this leaflet thing you should really increase your publicity in the media probably because the average person, no matter what his language will see television, and the advertisement should be at a time when they see that Eastenders, Big Brother and whatever you think, because a lot of people will understand that language. If a youngster in Eastenders has a problem then everybody will come. (GP FG25 medium screening practice)

Several practices suggested that including information on chlamydia screening and screening sites on practice websites would help to raise the profile of screening.

I think maybe advertising on the website and places that young people were going to look and read, as well as having the whole list of where they can get access to everything, not just one service. (Nurse FG19 low screening practice)

Several respondents commented that the promotional materials were also an important reminder for clinicians.

*Keep reminding us, keep bringing it up in meetings, trying to change the way you advertise and put the message across, always trying to think of new ideas*.

*Oh, no no we were saying chuck around a few of the posters*.

Also reminding clinicians, I think you have to keep on top of it really because I know our numbers dipped for a couple of months. (FG 2 medium tester respondent 3 and 4)

## Discussion

### Key findings

Although two-thirds of practices reported they were displaying posters and leaflets they were not prominently displayed in most practices. Only a minority of practices reported actively using the posters and leaflets on an ongoing basis, to raise the visual profile of chlamydia screening, in reception, waiting areas, lavatories and consulting rooms. Although about half the practices were using posters and leaflets, many of these had low rates of screening and were not backing up the advertising by routinely offering opportunistic screening to their target population. However, the study design does not allow us to say that the use of promotional materials alone will increase screening rates. It was interesting that some staff in many practices thought using posters and leaflets would cause offence or embarrassment to their patients. Staff thought this could be decreased by using more discrete posters and smaller leaflets or by using envelopes. Distribution of chlamydia leaflets by receptionists was thought to be inappropriate by some practices, as they thought patients would be offended when being offered a leaflet in public waiting areas or reception. Practices suggested other ways to promote chlamydia screening including a national advertising campaign and sending letters to patients' homes.

### Other work in this area

There is a paucity of literature exploring professionals' views about promotional posters and leaflets for chlamydia screening. Several studies exploring the use of promotional materials in other sexual health areas found that young patients are less likely to read posters than older patients [14-16]. These authors found that the public nature of waiting rooms and reception areas may inhibit patients collecting materials covering sexual health. Leaflets have been given to patients by GUM clinic receptionists to promote HIV testing [14,15]. Ivens and Sabin found that although patients' knowledge of HIV increased they were not more likely to accept a test compared to those offered a verbal discussion [15]. A recent general practice postal survey also shows that use of promotional materials covering sexually transmitted infections (STIs) is low in Australia - only one-fifth of GP respondents reported that they displayed posters covering STIs in their waiting room [17].

Andersen found a poor response to a multiple media campaign in Denmark which aimed to encourage chlamydia screening in young people [18]. Posters and leaflets in health, education and recreation centres, an internet web page, radio, television and newspaper interviews, were used to encourage young people to request a test kit for chlamydia. It resulted in a large proportion of requests for test kits from those ineligible to receive them [18], which indicates that leaflets need to be targeted at the at-risk population. This was done by several of the practices in our study.

It has previously been found that posters alone do not change the patient-professional interaction. In a US study, although 60% of patients noticed a poster campaign inviting patients in a US family practice clinic to discuss weight loss, the posters did not increase the proportion of patients reporting a change in patient-physician conversations about weight loss [19]. This work suggests that posters alone will not increase uptake of any health intervention without the willingness of health professionals to follow-up the advertising with offers to participate in screening.

Edwards *et al's *2003 systematic review of communicating individual risk in screening programmes suggests that communication understanding is associated with higher uptake of tests, although none of the studies included in this review used posters or patient leaflet [20]. The interventions described were risk appraisal questionnaires, or tailored printed materials and counselling. These authors point out that further evaluation of strategies is needed to promote informed decision- making and increase uptake of screening tests. O'Connor *et al*'s 1997 systematic review found that decision aids were better than usual care for patient facing screening or treatment decisions [21]. Written decision aids supported patients' decisions by making them feel better informed [21]. This study points out that decision aids increased preferences for some interventions (for example Hepatitis B vaccinations) but not others (dental surgery) through increasing patients' knowledge but variations exist in behaviour to accept screening or treatment.

### Strengths and weaknesses

This is the first study to elicit the views of general practice staff, using qualitative methods, about NCSP chlamydia posters and leaflets. We expect that other general practices would show similar attitudes and beliefs to those demonstrated by the staff in this study. We used a focus group approach. Although interviews may have allowed more junior members of the team and non clinical staff to speak more frankly about their views, we decided on the focus group setting as this is the usual setting for practice meetings and how the chlamydia screening coordinators approach the practice to discuss screening. Our perception was that all staff were given the opportunity to vocalise their attitudes to screening. The questions covering promotional materials formed part of the longer focus groups and, therefore, we may have obtained even more detailed data if we had concentrated on a single area, but it is likely that practices and many staff may have declined to participate if covering just this narrower topic. Although specific open questions were used about posters and leaflets, we did not show participants the NCSP leaflets or posters. EF has nursing and research training and has no direct involvement in the NCSP, so this allowed her to approach the work without any preconceptions. We were not able to obtain patients' views of NCSP promotional materials, as it was outside the scope of this study, but is the focus of future work. The study design did not allow us to say whether the use of promotional materials will increase chlamydia screening. As part of a multifaceted intervention, promotional materials were successfully used to increase chlamydia screening in North Carolina, USA [22].

### Implications of this research

#### Implications for the NCSP and chlamydia coordinators

The NCSP should consider developing specific posters and leaflets promoting chlamydia screening that are suitable for the general practice waiting room. The posters need to be eye-catching for young people but not offensive to the elderly or those with young children. Leaflets need to be developed in different formats to suit different scenarios in the general practice setting. Credit card sized discrete leaflets would be more suited to receptions and waiting room areas, whereas longer leaflets are more suitable for clinicians to distribute as screening is offered. As staff suggested, in areas of high ethnicity the NCSP should consider leaflets in different languages and leaflets should inform young people how often they should be screened. Chlamydia coordinators need to promote and audit the use and display of chlamydia screening promotional materials more actively across all general practices registered with the chlamydia screening programme. Coordinators need to emphasise to practice staff that leaflets and posters need to be followed up with a verbal offer of screening opportunistically whenever a 15-24 year old visits the practice. All practice staff will need training on how to approach this.

In summary, the NCSP and coordinators of the service at PCT level should:

Make posters more suitable for a general practice setting

Make posters acceptable to elderly patients who may also view them

Make leaflets less obviously about chlamydia so that they can be given to or picked up by patients without embarrassment

Produce pocket or credit card sized leaflets

In areas of high ethnicity, consider leaflets in different languages, or use more pictorial messages

Audit use and display of posters and leaflets

Ensure leaflets cover how often young people should be screened

Educate practice staff on how to follow up leaflets and poster use with offer of a chlamydia screen

#### Implications for practices

The NCSP needs to raise the profile of chlamydia screening in general practice. Practice staff need to discuss, with their screening coordinator, how the practice can promote chlamydia screening most effectively using promotional materials. The posters need to be displayed more prominently, so that they are more visible to young people, and practices should consider how they can increase distribution of leaflets through receptionists and clinicians. Practices need to review their use and display of sexual health promotion materials at least six monthly, as many professionals recognised that these lose their impact if not changed regularly.

Education to change practice staffs' attitudes towards sexual health is needed to reduce their worries and possible prejudices about displaying the chlamydia materials and following up with screening offers. A behavioural intervention approach using the Theory of Planned Behaviour [23] might be appropriate to address these issues. The promotional materials will be part of this approach to help normalise screening within the practice setting and make it more acceptable to staff and patients to offer screening opportunistically. Staff attitude to screening can also be influenced by education about the epidemiology and sequelae of chlamydia and the value of screening. Role play, videos or IT based materials can be used to increase practice staffs' confidence to be able to follow up poster displays and leaflets with verbal offers of chlamydia screening whenever they see a 15-24 year old patient. The education will need to be tailored for clinicians or the different staff; that is health care assistants and receptionists.

In summary, general practice staff should:

Increase awareness of all staff of NCSP and the posters and leaflets available

Display posters and leaflets more prominently

Display leaflets where they can be easily picked up by young people

Review display of leaflets and posters regularly, e.g. six monthly

Agree on when leaflets should be offered to patients by receptionists

Agree on when leaflets should be offered to patients by health-care assistants

Agree on when leaflets should be offered by patients by nurses and doctors

Order more discrete posters

Order smaller more discrete leaflets

Consider chlamydia envelopes if leaflets are not considered discrete enough for patients

Undertake role play or video based education on how staff can offer leaflets

Undertake role play or video based education on how staff can follow-up poster advertising in waiting room and patient leaflets with a screening offer

Consider postal invitations for chlamydia screening

## Conclusion

In conclusion the use of chlamydia screening posters and leaflets are not being optimised within most general practices. The NCSP posters need to be made eye-catching to the target group but acceptable to other patients visiting the practice. Leaflets need to be more discrete so that young people are happy to pick them up and read them. Clinicians need to follow-up poster advertising and leaflets with an offer of a chlamydia screen; video or role play based education may help them feel more confident to do this. To maintain their impact posters and leaflets need to be moved or changed six monthly and their use should be audited.

## Competing interests

Dr Cliodna McNulty writes the HPA Diagnosis of Chlamydia Quick Reference Guide for General Practices.

Isabel Oliver and William Ford-Young are members of the English National Chlamydia Screening Advisory Group; Sarah Randall is a former member.

## Authors' contributions

EF, RHJ, IO, SR, WFY, PB and CM contributed to the study design and writing of the protocol. EF, RHG & CM undertook the focus groups and analysed the data. EF and CM drafted the manuscript. RHJ, IO, SR, WFY and PB had input into the manuscript. All authors read and approved the final manuscript.

## Pre-publication history

The pre-publication history for this paper can be accessed here:



## Supplementary Material

Additional file 1 **Posters and leaflets available during the study period**. Click here for file
