# Supplementary material for: Promoting chlamydia screening with posters and leaflets in general practice - a qualitative study
Source: BMC Public Health. 2009 Oct 12;9:383. doi: 10.1186/1471-2458-9-383 (PMC2766388; doi:10.1186/1471-2458-9-383)

**Additional file 1**

**Title: Posters and leaflets available during the study period**

**Description: Leaflet used in consent procedure by the majority of general practices**


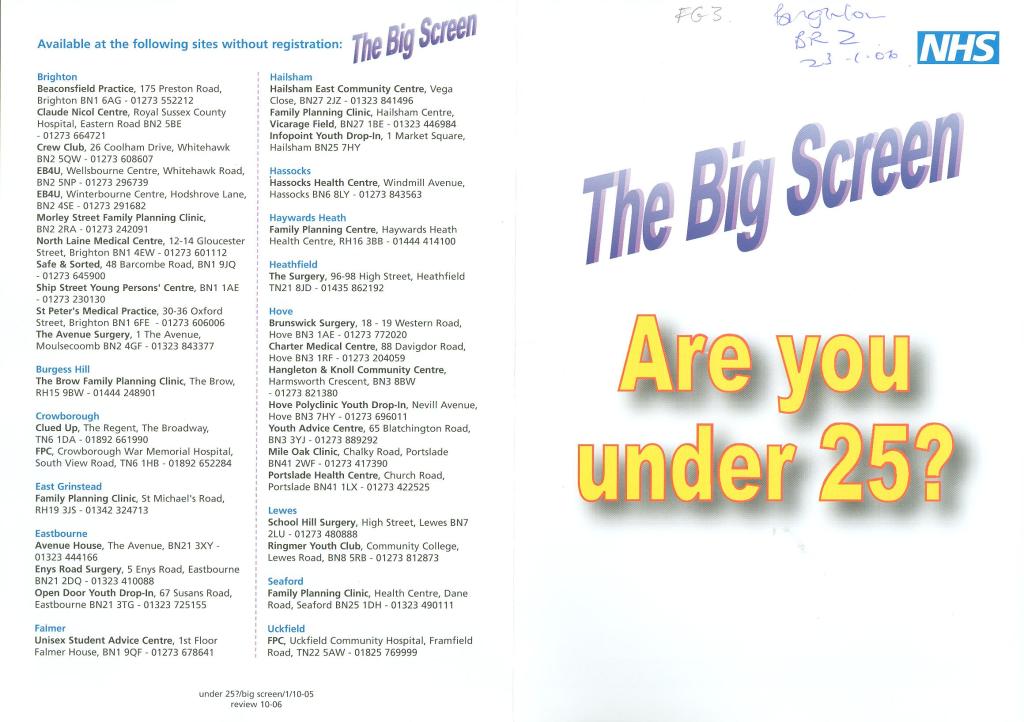


**
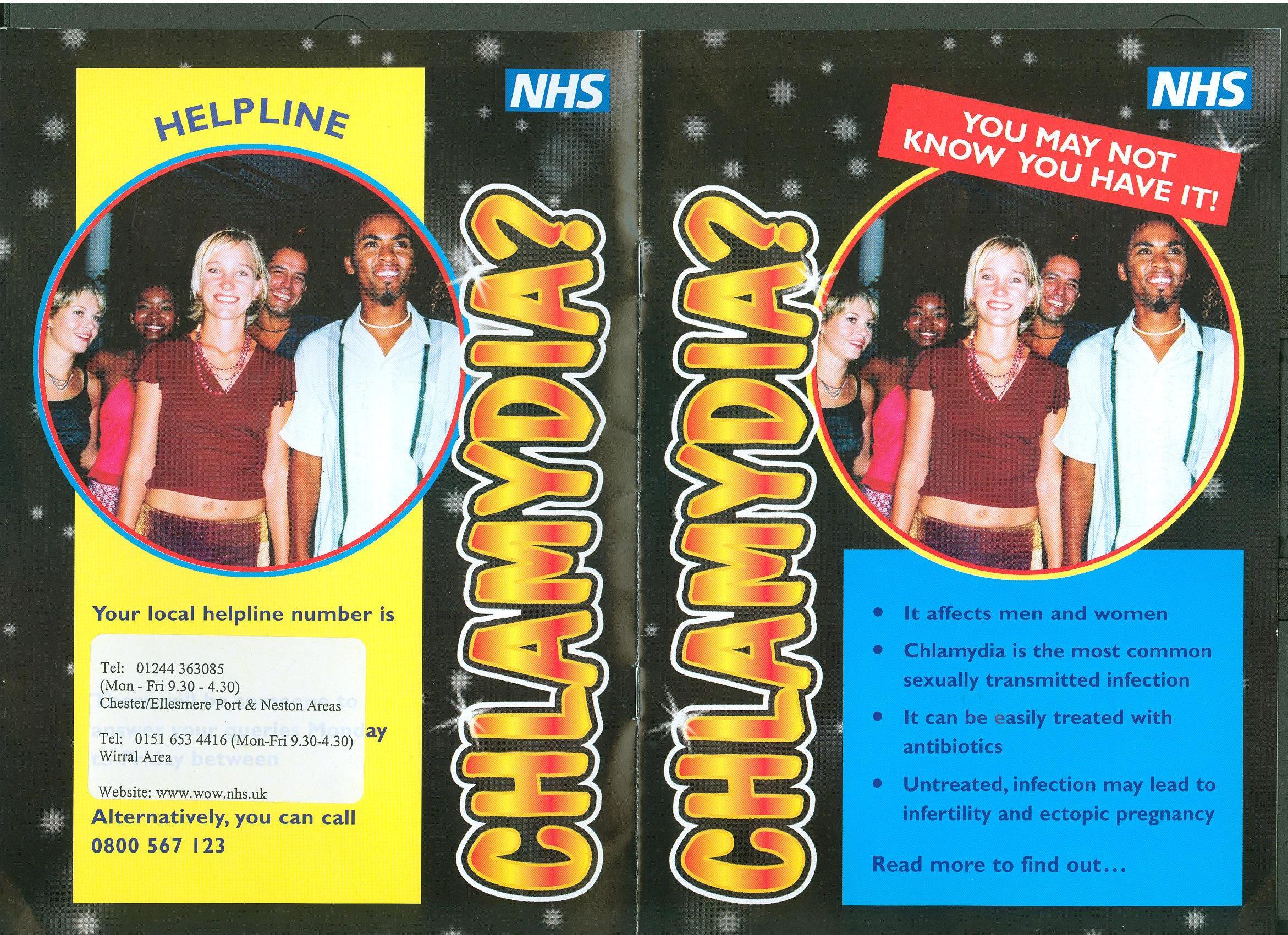
**

**Description: Department of Health posters used as part of the ‘Sex Lottery’ campaign to be used in a range of health care**
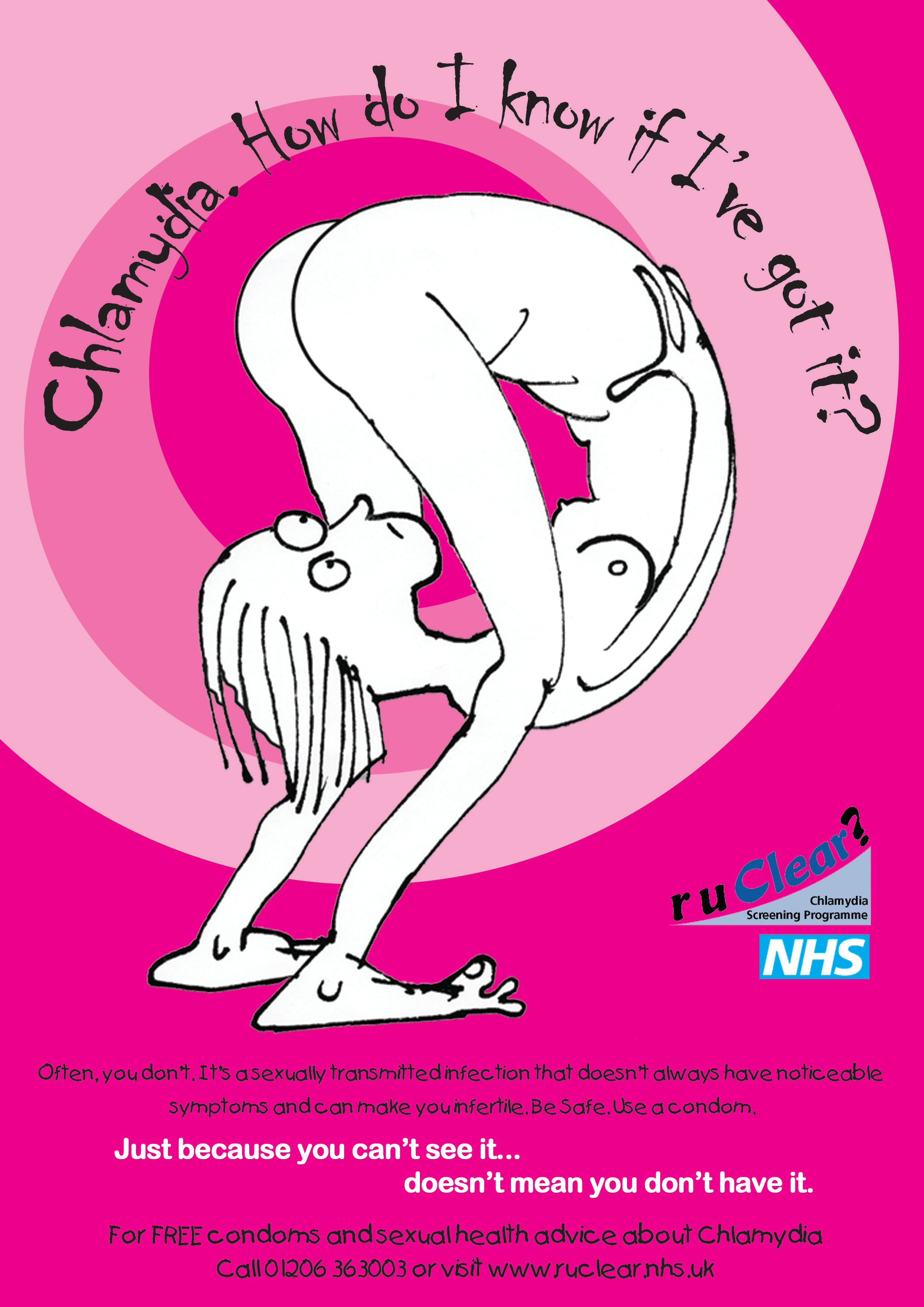
**settings**

**
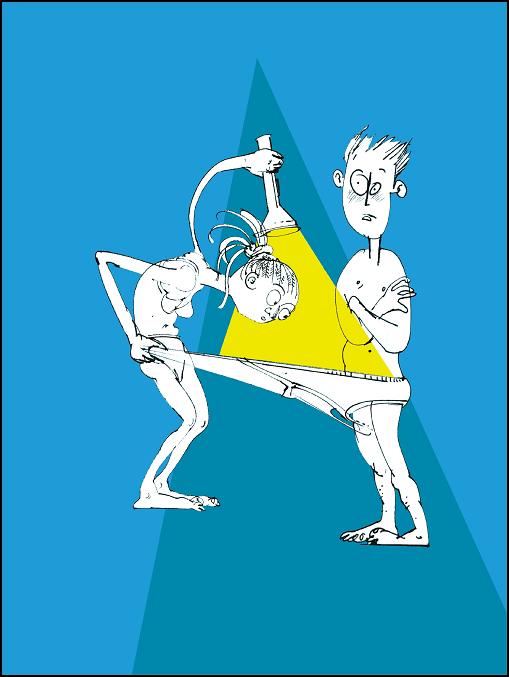
**

**Description: Sample of credit card-sized information distributed in one programme area**

**
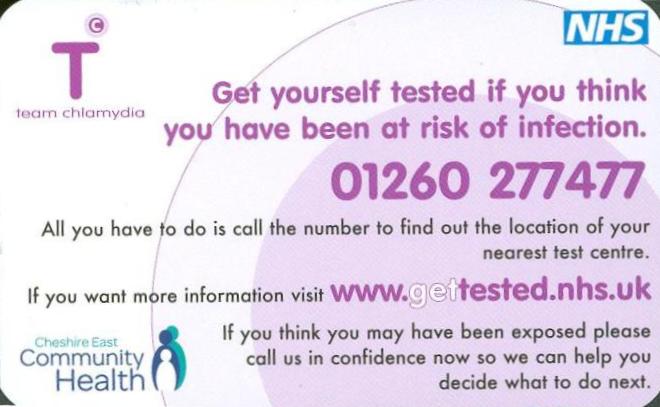
**

**
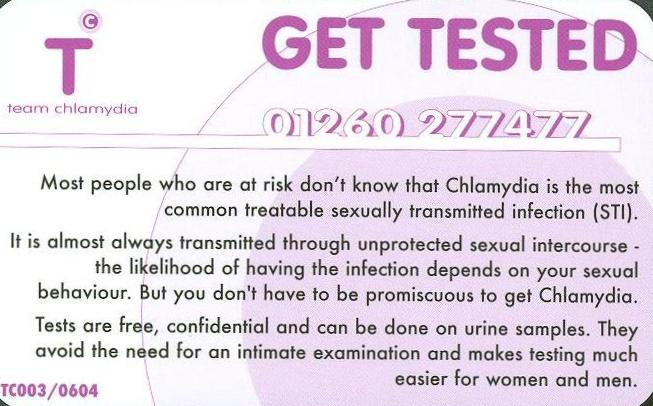
**

**Description: NCSP Poster available from 2003**

**Description: NCSP Poster available from 2006**


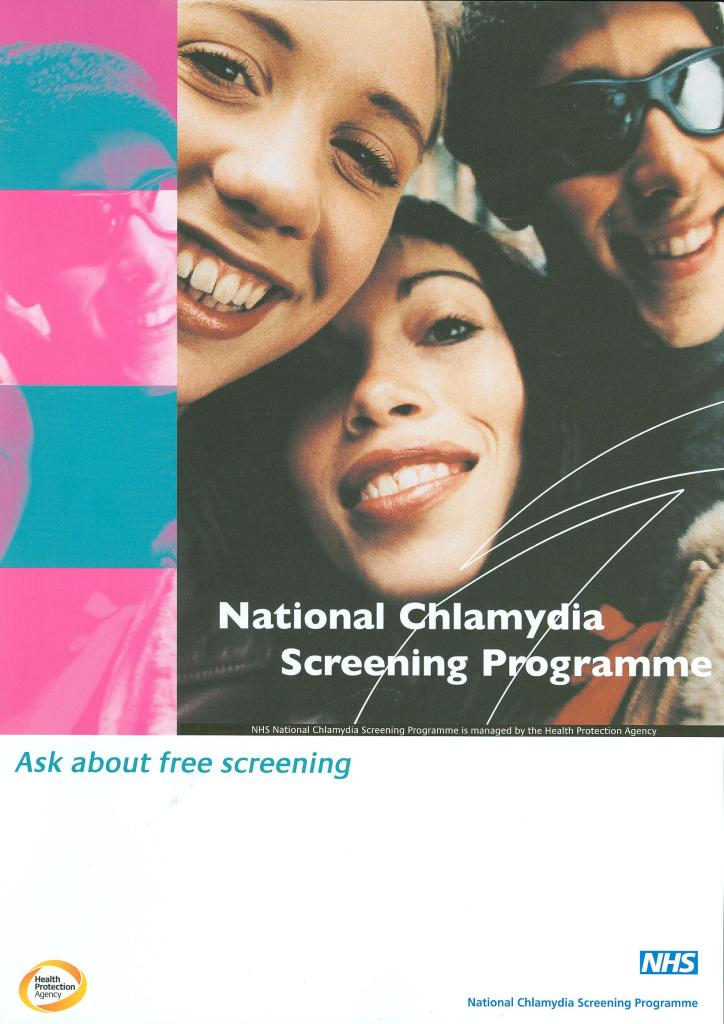

Supplement: Additional file 1 — Posters and leaflets available during the study period. [file 1471-2458-9-383-S1.DOC]
